# X-ray Fourier ptychography

**DOI:** 10.1126/sciadv.aav0282

**Published:** 2019-02-01

**Authors:** Klaus Wakonig, Ana Diaz, Anne Bonnin, Marco Stampanoni, Anna Bergamaschi, Johannes Ihli, Manuel Guizar-Sicairos, Andreas Menzel

**Affiliations:** 1 Paul Scherrer Institute, Forschungsstrasse 111, 5232 Villigen PSI, Switzerland.; 2 ETH and University of Zürich, Institute for Biomedical Engineering, 8093 Zürich, Switzerland.

## Abstract

To a large extent, the performance of imaging systems is determined by their objectives, which affect properties as varied as collection efficiency, resolving power, and image distortions. Such limitations can be addressed by so-called aperture synthesis, a technique used, for instance, in radar, astronomy, and, increasingly, microscopy. Here, we apply such techniques to x-ray imaging and demonstrate how Fourier ptychography can be used at transmission x-ray microscopes to increase resolution, provide quantitative absorption and phase contrast, and allow for corrections of lens aberrations. We anticipate that such methods will find common and frequent applications, alleviating a number of limitations imposed by x-ray optical elements, offering an alternative approach to phase contrast imaging, and providing novel opportunities to mitigate radiation damage.

## Teaser

A new image acquisition scheme increases resolution and sensitivity of x-ray microscopes.

## INTRODUCTION

In addition to the absence of distortions, an ideal imaging system ought to have three quintessential features: high resolution, high contrast, and a quantitative representation of the probed specimen. The achievable resolution is fundamentally related to the numerical aperture (NA) of the objective, which acts invariably as a low-pass filter. Whereas essentially perfect lenses allow microscopes to perform close to theoretical limits in the case of visible light, no equally powerful objectives are available for x-ray imaging. Consequently, the resolving power of x-ray microscopes scales distinctly less favorably than the drastic decrease of wavelengths may suggest, and somewhat paradoxically, instruments operating at shorter wavelengths, for which thicker and denser samples are translucent, typically yield lower resolutions than those operating at longer x-ray wavelengths.

X-ray optics can be classified in three categories: diffractive optics, such as Fresnel zone plates (FZPs), reflective optics, i.e., mirrors, and refractive optics, e.g., compound refractive lenses (CRLs). Despite recent progress reported in (*1*, *2*), mirrors and refractive optics often introduce distortions that render their use as objectives extremely challenging. Consequently, transmission x-ray microscopes (TXMs) most commonly use FZPs as objectives, which allow for high NAs and are less prone to aberrations but, at high x-ray energies in particular, are of limited efficiency only.

This has given rise to an intense interest in “lensless” x-ray imaging techniques, in which image formation is not achieved by optical elements but by mathematical “phase retrieval” algorithms (*3*). Of this set of techniques, ptychography has become particularly popular over the past decade (*4*). The method requires overlapping subsections of the sample to be illuminated with a coherent beam and diffraction patterns to be acquired at each position. Iterative algorithms are then used to reconstruct a complex-valued representation of the sample transmissivity and the illuminating wavefield (*5*–*7*).

Recently, a similar approach for lens-based imaging has been demonstrated for visible light. In this so-called Fourier ptychography (*8*, *9*), scanning the direction of illumination effectively scans the sample’s spatial-frequency content through the aperture of the objective. Using the same phase retrieval techniques results in a combination of the resolving power of high-NA objectives with large fields of view, typical of low-NA systems; moreover, both quantitative absorption and phase contrast can be reconstructed simultaneously. Last, analogous to the probe retrieval in “standard” ptychography (*5*, *6*), Fourier ptychography is able to reconstruct the pupil function, which allows a number of aberrations to be reliably corrected for (*10*).

All of these properties are highly desirable for x-ray microscopy, and in 2016 Simons and co-workers suggested the use of Fourier ptychography for x-ray imaging (*11*). Yet, to the best of our knowledge, no successful implementation of x-ray Fourier ptychography has been reported thus far.

## RESULTS

**Fig. 1:**
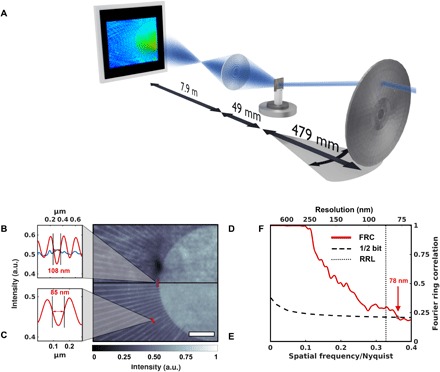
X-ray Fourier ptychography by scanning the illumination direction. (**A**) A sketch of the experimental setup. By inserting a movable pinhole (not shown) close to the fixed condenser, a standard TXM setup can be modified for Fourier ptychography as the change in illumination direction can be achieved by selecting individual subfields of the condenser. (**B** to **E**) A comparison of the flat-field corrected full-field image (D) and the magnitude of an x-ray Fourier ptychographic reconstruction (E) highlights the improvement in image quality and resolution. Scale bar, 2 μm. The test pattern’s last line cut indicates a linewidth of 150 nm, which is resolved with both techniques. Yet, the innermost lines, while being washed out in the full-field image, are resolved in the Fourier ptychographic reconstruction (B) (blue and red, respectively). Even smaller features with a width of 85 nm are visible in the Fourier ptychographic reconstruction (C). (**F**) A Fourier ring correlation (FRC) between two subsets of the Fourier ptychographic scan confirms the improvement in resolution. The dotted line marks the Rayleigh resolution limit (RRL).

The direct adaptation of the most common approach to Fourier ptychography requires the change of illumination direction while keeping any other changes of the illumination to a minimum. For this task, we took advantage of a peculiar condenser design found in TXMs, in which the standard FZP is replaced by an array of linear gratings and in which each subfield’s constant pitch preserves the subfield’s shape when diffracting toward a common “focus” (*12*, *13*). While this geometry is usually used to achieve a flat tophat-like illumination over the field of view, for the purpose of changing illumination direction, we inserted a 20 μm pinhole through which we could illuminate single subfields individually. Moving the pinhole, thereby selecting different subfields, acted in effect as translation of the spectrum in Fourier space (Fig. 1A). As an objective lens, we used an FZP with an outermost zone width of 70 nm, matching the NA of the condenser lens. Using 8.7 keV x-rays, i.e., a wavelength of 0.14 nm, we imaged a Au test pattern with a structure height of 1.5 μm.

The resolution in this chosen acquisition scheme remains limited by the combined NA of the condenser and objective; i.e., the frequency range over which the imaging system has any finite response is not fundamentally increased. In addition, this approach is rather sensitive to any structure in the illumination that may be caused by imperfections upstream of the condenser or by unwanted variations between condenser subfields. Yet, as Fig. 1 illustrates, the ptychographic reconstruction of 142 acquisitions of 3 s each allows for enhanced high-frequency content compared to a common full-field TXM image acquired over 10 s in the absence of the pinhole. As a common resolution metric, Fourier ring correlation (FRC) between two independent images of the Fourier ptychographic measurement was used to quantify the reproducibility of the frequency content of the sample’s spectrum (*14*). For this task, we split the scan into two sets of 71 acquisitions each, which we reconstructed independently. Choosing the threshold corresponding to an average information content of ½ bit per pixel, the FRC indicates an estimated resolution of 78 nm (Fig. 1F), well supported by line scans (Fig. 1, B and C). We have no indication that any image or reconstruction (Fig. 1, D and E, or even the individual input images to the FRC, not shown) should be considered dose-limited, but note that due to the pinhole, the flux during the ptychographic scan was reduced by more than three orders of magnitude.

**Fig. 2:**
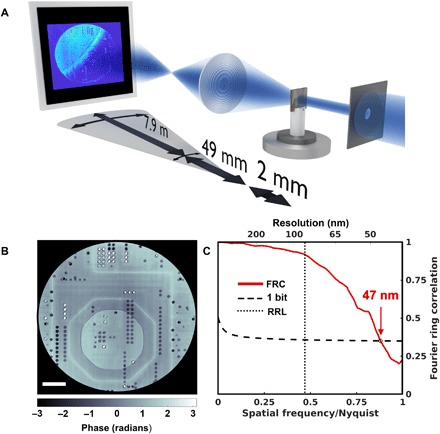
X-ray Fourier ptychography by scanning the objective lens. (**A**) A sketch of the experimental setup. Both objective lens and detector are scanned perpendicular to the optical axis. (**B**) The reconstructed phase image of an ASIC. Scale bar, 5 μm. (**C**) The resolution for a reconstruction using an FZP with an outermost zone width of 70 nm, a diameter of 100 μm, and a scan range of 80 μm was estimated using the Fourier ring correlation (FRC) between two independent scans to 47 nm, i.e., significantly below the Rayleigh resolution limit (RRL) of 85 nm for a conventional TXM, marked by the dotted line.

An alternative experimental realization, suggested also in (*11*), comprises scanning the objective lens and detector through the diffracted beam (Fig. 2A) (*15*). Not only does this approach avoid the complexity of keeping the illumination constant while changing the angle of incidence, but the maximum resolution is now limited by the maximum diffraction angle captured by the scan rather than by optical elements. The scan range extends the NA of the objective, in effect forming a synthetic lens and exceeding the NA of any available x-ray optics.

A plane-wave illumination was defined by a 30 μm pinhole close to the sample, which in this case was an application-specific integrated circuit (ASIC) (*16*). Because of the limited size of the detector, the movement of the objective lens had to be correlated with an adjustment of the detector position. To account for position uncertainties and for deviations in the magnification for different directions, the acquired raw data frames had to be aligned in a postprocessing step (*17*). A scan range of 80 μm for the FZP was chosen, corresponding to a synthetic lens with an outermost zone width of 39 nm and a diameter of 180 μm, thus improving the Rayleigh resolution limit from 85 to 47 nm.

**Fig. 3:**
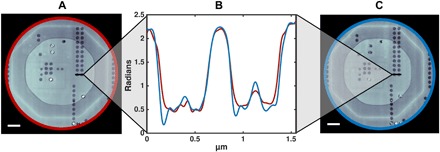
Confirmation of quantitativeness. (**A** and **C**) A reconstruction of the same ASIC, measured with Fourier ptychography (A) and conventional ptychography (C) (*16*). Scale bar, 2 μm. Resolution estimate for (A) is 47 nm and that for (C) is 41 nm (*16*). (**B**) The phase of the x-ray Fourier ptychographic reconstruction was compared to its conventional ptychographic counterpart. (**C**) The line cuts of both reconstructed phases reveal that the phase profiles match and thus a quantitative reconstruction can be provided (red for the Fourier ptychographic reconstruction and blue for “conventional” ptychography).

Fourier ptychography’s ability to yield phase information in absence of phase-shifting elements is demonstrated in Fig. 2B. Not only are halo artifacts, a common issue with Zernike phase contrast, absent in the reconstructed image, but the contrast is quantitative, as can be validated in comparison with an earlier, independent high-resolution measurement on the same microelectronic sample (Fig. 3) (*16*, *18*). The FRC yielded an estimated resolution of the Fourier ptychographic reconstruction of 47 nm (Fig. 2C).

The reconstructed pupil function shows a notable phase contribution, which can be interpreted as aberrations of the imaging system (fig. S3). They are conveniently expressed in terms of Zernike polynomials, $Z_{n}^{m}$. Related to freedom of gauge, polynomials with radial degree *n* = 0 or 1 do not correspond to image degradation and cannot be uniquely attributed to pupil function or sample spectrum. The contribution of $Z_{2}^{0}$, however, indicates that the sample image was not perfectly focused onto the detector. While this would have reduced image quality and hampered quantitative interpretation of the contrast in standard TXM mode, Fourier ptychographic reconstructions do not suffer such adverse effects (fig. S4).

## DISCUSSION

These measurements demonstrate how relatively simple modifications in data acquisition strategy can be used at conventional TXMs to enhance imaging capabilities in terms of resolution, contrast, and robustness against image distortions. The experimental complications that we encountered during these proof-of-principle measurements can mostly be addressed rather easily. Most cumbersome were limitations posed by the detectors, i.e., their limited size, which required their being repositioned when scanning the objective lens, slow readout when using charge-coupled devices (CCDs), or low-flux requirements when aiming for subpixel data interpolation (*19*). Yet, faster and larger detectors are, in principle, available and/or are being developed. Dedicated beamline optical elements and added control over the x-ray source can increase reliability and speed of scanning the illumination. Together with further robustness against illumination fluctuations (*20*), the ptychographic scan could then become part of standard acquisition schemes, unobtrusive to the experimenter.

Fourier ptychography allows high-resolution optics to be replaced by more efficient elements, even if by themselves they were limited to lower NAs or subject to aberrations (*1*, *21*). The technique could become useful for in situ or in operando measurements that require large working distances between sample and objective lens. Whenever the need for scanning permits, we see no fundamental obstacles precluding Fourier ptychography to enhance images or image modalities (*22*) for a multitude of full-field x-ray microscopes. Given that the brightness of available sources is expected to increase by orders of magnitude in the coming years (*23*), Fourier ptychography may be a promising way of taking advantage of the drastically increased coherence. At the same time, limited coherence requirements (*9*) allow Fourier ptychographic acquisition schemes also to be used at less brilliant sources, including laboratory-based systems. In regard to both development and use, we expect the technique’s availability to inform the choice between microscopes using objectives (*1*, *12*, *24*, *25*) and propagation-based techniques (*26*, *27*) and to be a valuable complement to existing imaging capabilities.

## MATERIALS AND METHODS

### Experimental setup

Experiments were carried out at the cSAXS beamline (X12SA) of the Swiss Light Source, Paul Scherrer Institut, Switzerland. Throughout the experiments, the photon energy was chosen to be 8.7 keV with a bandwidth Δ*E*/*E* ≈ 2 × 10^−4^. The source of size ~200 × 20 μm^2^ (horizontal × vertical) was approximately 34 m upstream of the sample, resulting in a coherence patch of ~24 × 240 μm^2^ (horizontal × vertical).

### Measurement technique I

To acquire a full-field TXM image, the beam illuminated a condenser lens with a diameter of 1 mm, a subfield size of 50 μm, and an outermost zone width of 70 nm (fig. S1A) (*13*), which results at the wavelength of 0.14 nm in an NA of 10^−3^. To block the direct beam, a 150 μm central stop was mounted on the same stage, close to the condenser. An order-sorting aperture was inserted to restrict the illumination of the sample to the first-order diffraction of the condenser. As the objective lens, an FZP with a diameter of 100 μm and an outermost zone width of 70 nm, matching the NA of the condenser lens, was placed 49.5 mm downstream of the sample. Each optical element was mounted on an assembly of stepper motors to allow for three-dimensional (3D) movements.

At a distance of 7.3 m, behind a He-filled flight tube of 7 m, the images were collected with a Photonic Science VHR Image Star x-ray camera, based on a full-frame Kodak CCD with an optical resolution of 4 μm. This geometry resulted in a magnification of 150 and an effective pixel size of 27 nm in the sample plane. Of the 3056 × 3056 pixels, we selected a readout size of 470 × 392 pixels, matching the illuminated region of interest. For the full-field image shown in Fig. 1D, 10 acquisitions with an exposure time of 1 s were taken and summed up. To account for inhomogeneities in illumination or detection efficiency, images were divided by acquisitions without sample.

To select single condenser subfields for Fourier ptychographic measurements, a 20 μm pinhole was inserted approximately 80 mm upstream of the condenser. As the scan pattern was to match subfield centers, the movement of the pinhole had to be carefully aligned to the condenser. For this task, we acquired a transmission map by scanning the pinhole across the condenser (fig. S1B). Assuming that the highest intensities occur at the center of the subfield, we acquired a 3 s frame at each such local maximum. A total of 142 acquisitions were used for the Fourier ptychographic reconstruction.

### Reconstruction

To lessen artifacts in the raw data related to the camera’s fiber coupling (fig. S1C), acquisitions were intensity-thresholded and pixels below this threshold were replaced by nearest-neighbor interpolation.

The reconstruction was performed using a MATLAB-based package and high-performance reconstruction engines written in C++, which were developed by the Coherent X-ray Scattering Group, Paul Scherrer Institute, Switzerland. The difference map algorithm (*5*) converged after 2000 iterations and was followed by 150 iterations of likelihood maximization (*7*). The reconstructed spectrum was backpropagated to direct space.

To estimate image resolution using FRC, the data were split into two disjoint sets, which were analyzed independently. Having thereby divided the flux by two, we compared the FRC to the ^1^/_2_-bit criterion (*14*).

### Measurement technique II

In a second experiment, the condenser lens, the central stop, and the order-sorting aperture were removed and replaced by a 30 μm pinhole close to the object plane, defining the field of view. The same objective lens as was used in the previous experiment created a magnified image of the sample at a distance of 7.9 m, where the direct beam was blocked by a 1.5 mm conical stainless steel central stop mounted on a 13 μm Kapton foil. As the required precision of the objective movements scales with the resolution, the objective was mounted on a 3D piezo-electric stage with a maximum range of 100 μm. The data were collected using a 400 × 400 pixel prototype MOENCH detector with a physical pixel size of 25 μm (*19*, *28*). Using slits 22 m upstream of the sample, the flux was reduced such that single-photon events were detectable within 0.25 ms acquisitions to allow for data interpolation to a pixel size of 6.25 μm. Incidentally, the use of the slits resulted in approximately the same flux at the sample position as for measurement technique I, which used a smaller pinhole and condenser, and increased the horizontal coherence length to about 150 μm.

Per scan point, 15,000 acquisitions were taken, which resulted in a total acquisition time of around 15 s per point including overhead. The FZP was scanned following a Fermat spiral (*29*) with an average step size of 3.5 μm and a diameter of 80 μm, resulting in 522 points. To follow the sample image, which moves during the scan of the objective lens, the detector was mounted on a hexapod and synchronously translated with micron precision according to the magnification of 160.

To avoid systematic artifacts in the reconstruction that could distort the resolution estimate by FRC, a second Fermat’s scan was performed, with an average step size of 5 μm for the FZP while keeping the same overall scan range of 80 μm. At each of the 255 scan points, 30,000 acquisitions of 0.25 ms were taken. Since we could use two independent measurements, we compared the FRC to the 1-bit criterion (*14*).

### Reconstruction

The data interpolation on the detector (*19*) introduced periodic artifacts (fig. S2), which were addressed by setting the contribution of the corresponding frequencies to zero. To account for a directional dependence of the magnification, an alignment routine had to be implemented. Since this registration is hampered by the direct beam impinging on the detector, we discarded the inner part of the scan pattern, i.e., within 25 μm from the center. In the case of the first scan, this reduced the number of scan points used for analysis to 362; in the case of the second scan, the number of scan points was reduced to 177.

Since frames differ in spatial-frequency content, the alignment (*17*) was performed sequentially on frames with substantial “overlap” in the frequency domain. Specifically, we defined a sequence of images with slowly varying frequency content and aligned each frame to the preceding one. Error accumulation was counteracted by repeating the process in reverse order, which was sufficient for convergence. More general repositioning schemes for ptychographic scans can be found in the literature [e.g., (*20*)] but were not found to further improve reconstruction quality. In the case of large detectors or sufficiently precise detector movement, this alignment process could be reduced to an affine-matrix search and thus would be independent of the scan parameters.

The Fourier ptychographic reconstruction took 600 iterations of the difference map to converge (*5*). The solution was then refined by 1800 iterations of likelihood optimization (*7*), before the reconstructed spectrum was backpropagated to direct space. Two coherent modes were reconstructed for the pupil function (*30*), the first of which is shown in fig. S3. We discarded the second mode, containing approximately 33% of energy and displaying essentially no structure, as background contribution.

## Supplementary Material

http://advances.sciencemag.org/cgi/content/full/5/2/eaav0282/DC1

## SUPPLEMENTARY MATERIALS

Supplementary material for this article is available at http://advances.sciencemag.org/cgi/content/full/5/2/eaav0282/DC1 Fig. S1. Condenser lens. Fig. S2. Raw data interpolation. Fig. S3. Reconstructed pupil function. Fig. S4. Summed projections without and with lens correction.
