# Supplementary material for: X-ray Fourier ptychography
Source: Sci Adv. 2019 Feb 1;5(2):eaav0282. doi: 10.1126/sciadv.aav0282 (PMC6358315; doi:10.1126/sciadv.aav0282)
Supplement: http://advances.sciencemag.org/cgi/content/full/5/2/eaav0282/DC1 [file supp_5_2_eaav0282__index.html]

Science Advances | Science Advances

## Supplementary Materials

**This PDF file includes:**

- Fig. S1. Condenser lens.
- Fig. S2. Raw data interpolation.
- Fig. S3. Reconstructed pupil function.
- Fig. S4. Summed projections without and with lens correction.

Download PDF

**Files in this Data Supplement:**

- Adobe PDF - aav0282\_SM.pdf
