# Supplementary material for: X-ray Fourier ptychography
Source: Sci Adv. 2019 Feb 1;5(2):eaav0282. doi: 10.1126/sciadv.aav0282 (PMC6358315; doi:10.1126/sciadv.aav0282)
Supplement: http://advances.sciencemag.org/cgi/content/full/5/2/eaav0282/DC1 [file aav0282_SM.pdf]

Science Advances
AAAS

advances.sciencemag.org/cgi/content/full/5/2/eaav0282/DC1

Supplementary Materials for

X-ray Fourier ptychography

Klaus Wakonig*, Ana Diaz, Anne Bonnin, Marco Stampanoni, Anna Bergamaschi, Johannes Ihli, Manuel Guizar-Sicairos, Andreas Menzel*

*Corresponding author. Email: klaus.wakonig@psi.ch (K.W.); andreas.menzel@psi.ch (A.M.)

Published 1 February 2019, *Sci. Adv.* **5**, eaav0282 (2019)
DOI: 10.1126/sciadv.aav0282

This PDF file includes:

Fig. S1. Condenser lens.
Fig. S2. Raw data interpolation.
Fig. S3. Reconstructed pupil function.
Fig. S4. Summed projections without and with lens correction.

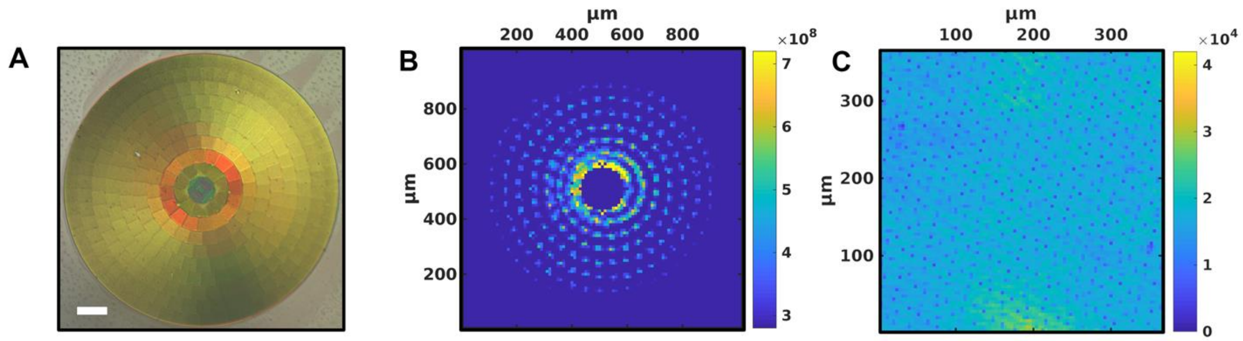

Fig. S1. Condenser lens. A, The condenser lens as used in standard transmission X-ray microscopes. The 1 mm condenser with an outermost zone width of 70 nm comprises 321 subfields, each with a width of 50 µm. Scale bar: 150 µm. This design allows for a Fourier ptychographic measurement by scanning a pinhole, which selects a single subfield containing a linear grating. **B,** To alleviate difficulties in the alignment of the pinhole with respect to the condenser, an intensity map was measured by scanning the pinhole. Local intensity maxima were chosen as scan points for the Fourier ptychographic data acquisition. **C,** The used detector suffers from artifacts related to its fiber coupling, i.e. visible dots in the flat-field. To retrieve the location of these distortions, a threshold-based mask was used. Once the pixel coordinates were known, a nearest-neighbor interpolation was applied.

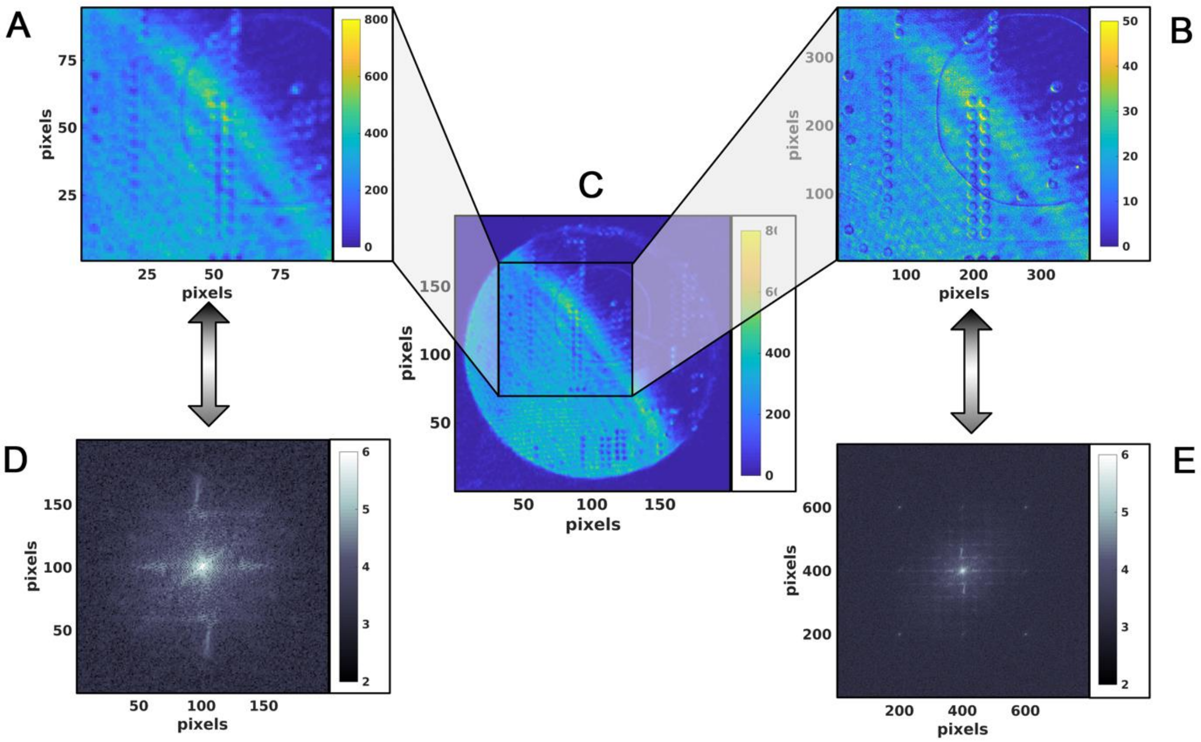

Fig. S2. Raw data interpolation. A–C, The MOENCH detector raw data with a pixel size of 25 µm **(C)** was interpolated to a 6.25 µm pixel size **(B)**. A direct comparison between a zoomed in raw data frame **(A)** and the interpolated image **(B)** highlights the improvement in resolution. **D,E,** Interpolation artifacts are visible as bright dots in the Fourier spectrum **(E)** absent in the raw data's spectrum **(D)**. To mitigate such high-frequency distortions, a mask was applied to the raw data's spectra before starting the Fourier ptychographic reconstruction.

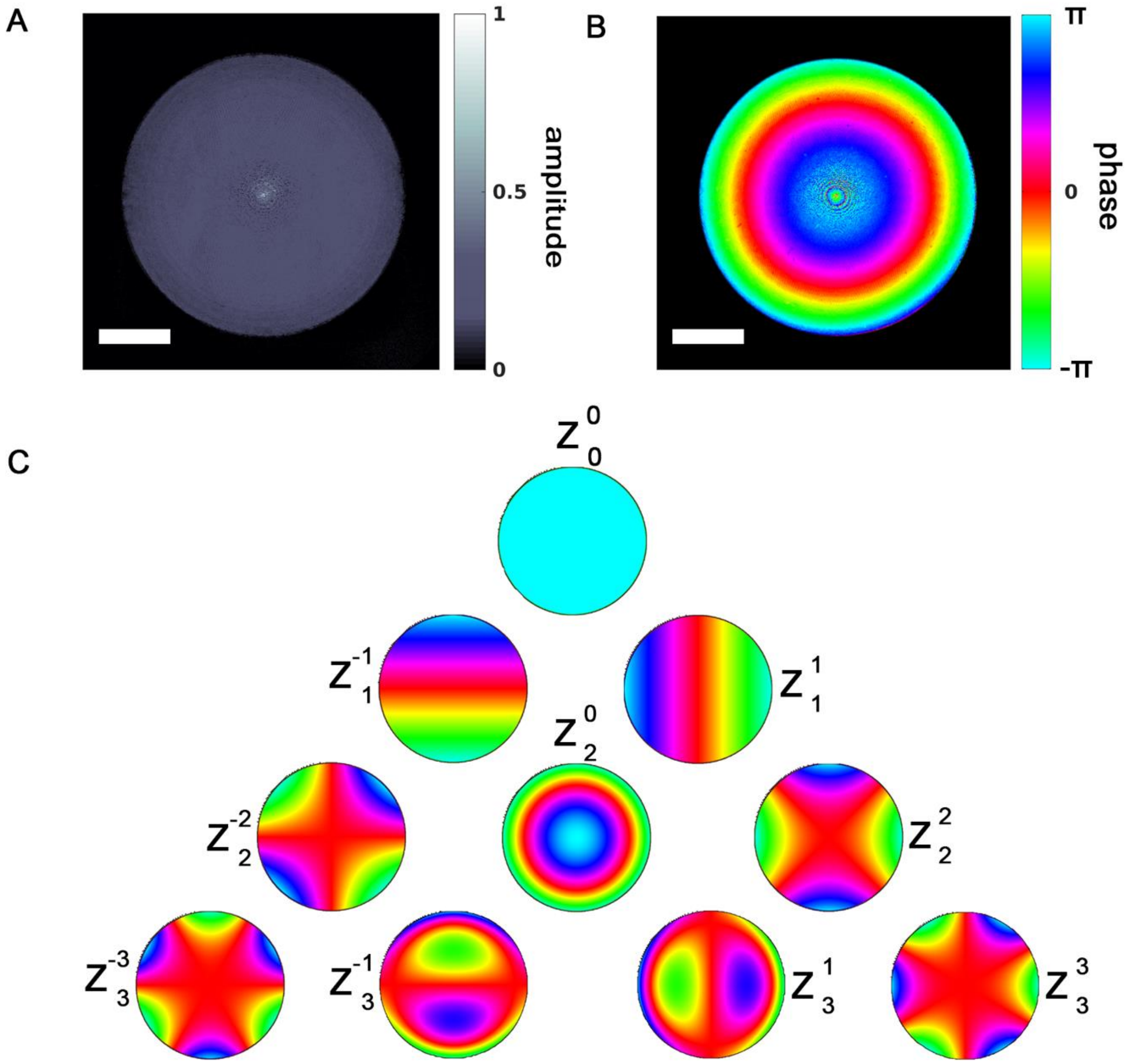

Fig. S3. Reconstructed pupil function. A,B, By reconstructing the pupil function and the sample spectrum at the same time, aberrations of the imaging system can be corrected as an additional phase factor in the pupil function. The amplitude of the complex-valued pupil is shown in **(A)**, its phase in **(B)**. **C,** If expanded in terms of Zernike modes, the phase **(B)** reveals a significant contribution of Z_2^0, which indicates that the objective was placed out of focus. For improved visibility, a mask with the shape of the objective was applied to **(B)**. Scale bar: 25 μm.

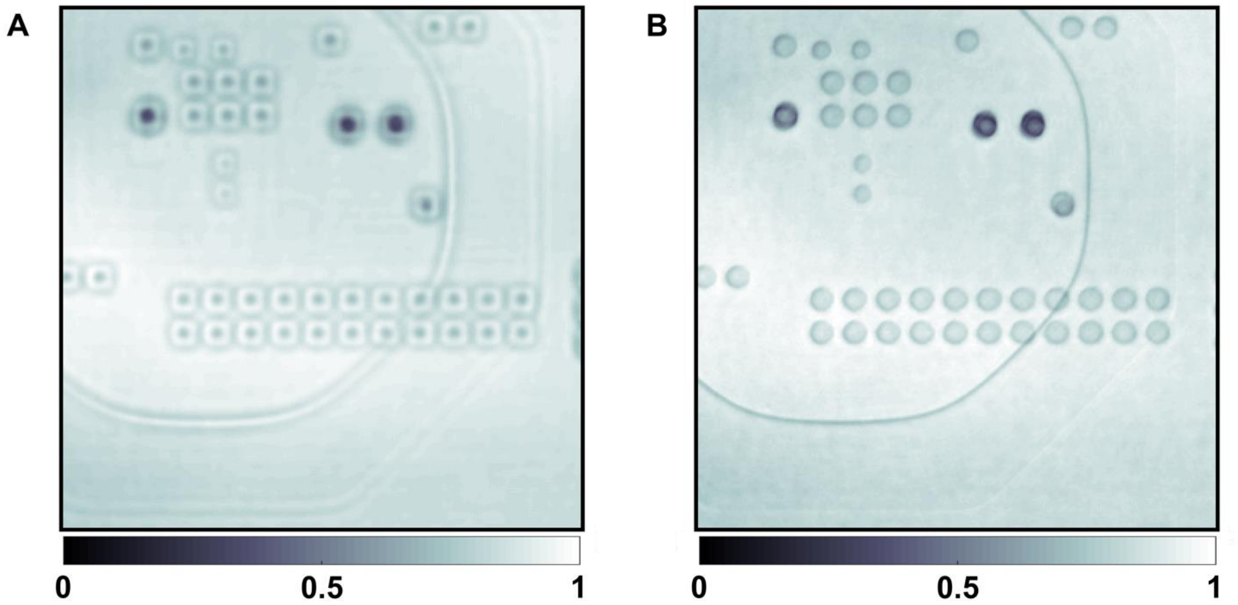

Fig. S4. Summed projections without and with lens correction. To assess the influence of the lens retrieval, ptychographic projections of the reconstructed spectrum were propagated to the detector plane without any lens aberration corrections **(A)** and using the reconstructed pupil function **(B)**. The projections were summed up for better statistics. Defocus is clearly visible in (**A).**
